# Gallic acid diminishes pro-inflammatory interferon-γ- and interleukin-17-producing sub-populations in vitro in patients with psoriasis

**DOI:** 10.1007/s12026-023-09361-9

**Published:** 2023-02-09

**Authors:** Sotirios G. Tsiogkas, Konstantina Apostolopoulou, Athanasios Mavropoulos, Maria G. Grammatikopoulou, Efthimios Dardiotis, Efterpi Zafiriou, Dimitrios P. Bogdanos

**Affiliations:** 1 Department of Rheumatology and Clinical Immunology, Faculty of Medicine, School of Health Sciences, University of Thessaly, Larissa, Greece; 2 Department of Neurology, Faculty of Medicine, School of Health Sciences, University of Thessaly, Larissa, Greece; 3 Department of Dermatology, Faculty of Medicine, School of Health Sciences, University of Thessaly, Larissa, Greece

**Keywords:** PBMCs, Flow cytometry, Th17, Th1, NK, NKT

## Abstract

Psoriasis is an inflammation of the skin mediated via the IL-23/Thl17/IL-17 pathway. We have previously demonstrated that the anthocyanin delphinidin diminishes in vitro the IL-17 and IFN-γ production of peripheral monocytes isolated by psoriasis patients (PBMCs). The degradation product of delphinidin is gallic acid (GA). This phenolic acid compound found in fruits, red wine, or green tea exerts pleiotropic antioxidant, anticarcinogenic, antimicrobial, and anti-inflammatory properties. Previous research has demonstrated the inhibitory effect of GA on pro-inflammatory transcription factors, such as STAT3, RORγt, and NF-κB, or cytokines as IL-1β and TNF, which contribute to psoriasis development. We investigated the effect of GA in vitro on PBMCs, which were stimulated ex vivo, from 40 individuals (28 diagnosed with psoriasis vulgaris and 12 healthy controls (HCs)). In our experiments, PBMCs were cultured untreated or were activated in the presence of phorbol 12-myristate 13-acetate/ionomycin with or without GA. We utilized multicolor flow cytometry to assess the production of inteleukin-17 (IL-17) and interferon-γ (IFN-γ) in T and NK cells. GA did not alter the fractions of IL-17- or IFN-γ-producing T and IFN-γ-producing NK cells in HCs. However, in psoriasis patients, the effect of GA on that cell population was significant. Specifically, GA decreased the frequency of IL-17-producing cells within the CD3^+^ (T) and CD3^+^CD4^+^ (Th) compartment; the frequency of IFN-γ-producing cells within the CD3^+^, CD3^+^CD4^+^, and CD3^+^CD4^−^ (Tc) compartment, and the frequency of IFN-γ-producing cells within the CD3^−^CD56^+^ (NK) compartment. Whether GA’s effect also appears in vivo needs to be investigated in future.

**Supplementary Information:**

The online version contains supplementary material available at 10.1007/s12026-023-09361-9.

## Introduction

Psoriasis is characterized by chronic inflammation of the skin. The interleukin (IL-)-23/T helper (Th) l17/IL-17 pathway has been recognized as crucial for the development of the disease. Skewing of CD4^+^ T cells towards a Th17 phenotype is in part mediated by tumor necrosis factor (TNF-) α and is characterized by signal transducer and activator of transcription 3 (STAT3) and retineic-acid-receptor-related orphan nuclear receptor gamma (ROR-γt) transcription factor upregulation [1]. Increased IL-17 production stimulates pro-inflammatory mediators in IL-17-R-bearing cell types, like keratinocytes. Another crucial component of the IL-17-mediated induction of psoriasis has been proposed to be nuclear factor kappa-light-chain-enhancer of activated B cells (NF-κB) [2]. Moreover, in psoriasis, additional cell subsets, like Th1 or Th1-like Th17, have been found in increased fractions compared to HCs [3]. Interferon-γ (IFN-γ), which is produced by such cell populations [4], has been suggested to induce a transcriptomic signature similar to that of psoriatic skin in unaffected skin [5].

We have previously demonstrated that delphinidin, which is an anthocyanin, effectively diminished in vitro IL-17 and IFN-γ production by phorbol 12-myristate 13-acetate (PMA)-activated peripheral blood mononuclear cells (PBMCs) isolated by psoriasis patients [6]. The degradation product of delphinidin has been found to be a phenolic acid, namely gallic acid (GA) [7, 8]. GA is a phenolic acid compound [9], detected both free and as part of hydrolysable tannins, widely in fruits (i.e., pomegranate) and vegetables [10]. It is also found in red wine or green tea [11]. Biologic properties of non-flavonoid polyphenols such as phenolic acids, ranging from antioxidant, anticarcinogenic, to antimicrobial or anti-inflammatory, have been presented in numerous studies (reviewed by [12]). GA has also been examined for similar activities [13–16]. A plethora of patented applications of GA and its esters in medicine has been reported. Indeed, various properties in industry as an anti-oxidant, an anti-angiogenic, or a cosmetic agent have been attributed to GA [11]. Intriguingly, the anti-oxidant activity of GA, when compared to other known anti-oxidant compounds, has been reported to be one of the highest [17, 18]. On the other hand, both GA and its esters have been reported to induce cell death in tumor cell lines [19, 20] suggesting that GA may have both anti-oxidant and pro-oxidant capacities [13]. GA’s anticancer activity has thus been investigated in many studies [21–23]. Further beneficial properties of GA include anti-microbial and anti-viral activities [13].

Based on the aforementioned observations regarding the significant in vivo or in vitro effect of GA, an investigation of the modulation of PBMCs isolated by psoriasis patients by GA was considered of particular interest. Of relevance to the present study, mounting evidence also supports the immunomodulatory and anti-inflammatory properties of GA. In murine models, researchers showed that GA treatment significantly inhibited NF-κB activation. Moreover, GA treatment compared to mock treatment resulted in lower levels of interleukin IL-1β and TNF-α expression in the small intestine and also lower levels of both cytokines in mouse sera [24]. Furthermore, in a murine chronic constriction injury model, GA reduced the levels of TNF-α, NF-kB, and phosphorylated STAT 3 (p-STAT3) in mouse sera [25]. Intriguingly, in an allergic rhinitis mouse model, GA treatment alleviated symptoms. Moreover, GA treatment suppressed levels of IL-17 and Th17 transcription factor, ROR-γt in the nasal lavage fluid [26]. The inhibitory effect of GA on pro-inflammatory molecules has also been investigated in vitro. Suppression of NF-κB phosphorylation has also been reported in LPS-challenged IPEC-J2 cell cultures when pre-treated with GA. Importantly, GA also abrogated TNF-α and IL-8 gene expression in IPEC-J2 cells [27]. In another study, poly (GA)—a compound enzymatically produced by GA—pre-treatment reduced IL-1β and IL-6 levels in supernatants of PMA-stimulated THP-1 cell cultures [28]. In A549 lung cancer cell cultures, GA treatment resulted in attenuated phosphorylation of both JAK and STAT3 [29]. In a HT-29 cell line culture that was challenged with human recombinant IL-17 and TNF-α, IL-17A expression was significantly suppressed when cells were co-cultured with GA [30].

In view of the above, we were interested to assess the in vitro effect of GA in an immune-mediated disease, using psoriasis as a reference disease. Due to the high instability of delphinidin, we intended to investigate whether the in vitro or in vivo anti-inflammatory effects exerted by delphinidin could potentially be expanded by the subsequent activity of GA after delphinidin’s rapid degradation. Researchers have already supported that both delphinidin’s metabolites and GA promote Treg differentiation when co-cultured with CD4^+^ naïve T cells isolated from mice [7]. However, the immunomodulatory effect of GA on PBMCs from individuals diagnosed with psoriasis has to our knowledge not been explored. The lack of sufficient information regarding this matter has led us to investigate by multicolor flow cytometry the effect of GA on IL-17 and/or IFN-γ expression by PMA-stimulated PBMCs isolated by individuals with psoriasis and healthy controls (HCs).

## Materials and methods

### Patients

Twenty-eight consecutive patients diagnosed with moderate to severe plaque psoriasis (Psoriasis Area Severity Index > 7) that attended the Outpatient Department of the Department of Dermatology of General University Hospital of Larissa and 12 HCs were enrolled in this study. Main demographic and clinical parameters of the enrolled participants are summarized in Table 1. Our patient cohort was consisted of 9 females and 19 males, with a mean age of 49 ± 10.6 years. In total, 8 individuals were naïve to any therapy, 5 were receiving conventional disease-modifying anti-rheumatic drugs, 7 were receiving apremilast, a PDE4 inhibitor, and 8 were receiving biologic agents (anti-IL-23, anti-IL-17) during blood sample collection. Clinical or radiological signs suggesting a psoriatic arthritis diagnosis were not apparent to any of the psoriasis patients. Written informed consent was signed by all subjects involved in this study, in accordance with the revised Declaration of Helsinki.

**Table 1:** Major demographic and clinical parameters of enrolled individuals

	Psoriasis patients (*n* = 28)	HCs (*n* = 12)
Males/females	19/9	8/4
Mean age ± SD (years)	49 ± 10.6	46.17 ± 13.3
Mean PASI ± SD	13.3 ± 8.67	
Naïve to therapy	8/28	
Receiving anti-IL-17 biologic treatment	4/28	
Receiving anti-IL-23 biologic treatment	4/28	
Receiving apremilast treatment	7/28	
Receiving cDMARD treatment	5/28	

*HCs* healthy controls, *SD* standard deviation, *PASI* psoriasis area and severity index, *IL-17* interleukin-17, *TNF* tumor necrosis factor, *cDMARD* conventional disease-modifying anti rheumatic drug

### Chemicals

GA (purity ≥ 98%) was purchased from Cayman Chemical (Ann Arbor, Michigan, USA). PMA and ionomycin were obtained from Sigma-Aldrich (Merck KGaA, Darmstadt, Germany). Brefeldin was obtained by GolgiPlug™, BD Biosciences. Each reagent was dissolved in dimethylsulfoxide (DMSO) and subsequently aliquoted to be stored at − 20 °C. However, final DMSO concentration never exceeded 0.1% in any cell culture experiment. Selection of the GA concentration used in this study was based on dose–response experiments utilizing GA at a concentration range from 5 to 100 μg/mL. Opted concentration was in accordance with published in vitro or in vivo studies on pharmacokinetics and bioavailability.

### Peripheral blood mononuclear cell isolation and cryopreservation

Heparinized syringes were used to obtain peripheral blood samples (20 mL) by venipuncture. Layering over the surface of a discontinuous LymphoPrep gradient (Axis-Shield, Oslo, Norway) and subsequent centrifugation were utilized to isolate PBMCs from each blood sample. A protocol involving two washings of isolated PBMCs with RPMI 1640 (GIBCO™, Thermo Fisher Scientific, Waltham, MA, USA) was applied afterwards. Cells were then enumerated using a Neubauer hemocytometer. Moreover, cell viability was assessed for each sample by trypan blue exclusion dye and routinely surpassed 95%. A cell cryoprotective solution consisting of 10% DMSO, 30% RPMI 1640, and 60% fetal calf serum (FCS) was produced. PBMCs were then re-suspended in that medium and aliquoted into cryotubes (Corning™, Thermo Fisher Scientific, Waltham, MA, USA). Aliquots were kept at − 80 °C for 24 h, and then were stored in liquid nitrogen containers until further use.

### Cell thawing

Cryotubes were removed from the liquid nitrogen containers and immediately transferred into a water bath of approximately 37 °C. Each cryotube stayed within bath for < 1 min and was subsequently transferred in a flow laminar hood. Cells and pre-warmed RPMI 1640 were simultaneously transferred dropwise in a centrifuge tube. Thawed PBMCs were then centrifuged for 5 min. Supernatant was afterwards decanted. Finally, PBMCs were re-suspended in a culture medium containing 10% FCS and 90% RPMI 1640.

### Cell culture

PBMCs were seeded in 6-well culture plates (Sigma-Aldrich, Merck KGaA, Darmstadt, Germany). In each well, 6 × 10^6^ PBMCs were placed within 3 mL of the culture medium. PBMCs were incubated at 37 °C (5% CO_2_). GA was added to PBMC culture 0.5 h prior to activation at an end concentration of 30 μg/mL. PBMCs were stimulated in the presence of PMA (50 ng/mL), ionomycin (1 μg/mL), and brefeldin A for 5 h. Cells that were not stimulated were used as controls, while cells supplemented only with GA were used as vehicle controls.

### Flow cytometric phenotypic analysis

A Guava EasyCyte 8 (Merck-Millipore, Burlington, USA) benchtop flow cytometer was utilized to explore PBMC sub-populations. Assessment of cell phenotypes and enumeration of subsets was accomplished using standard anti-human fluorochrome-conjugated monoclonal antibodies targeting specific surface markers, as previously described [31]. Briefly, identification of T cell subsets involved use of fluorescein isothiocyanate–conjugated anti-CD3 (clone UCHT1), phycoerythrin (PE)-conjugated anti-CD56 (clone HCD56), and (PE-Cy7)-conjugated anti-CD4 (clone RPA-T4). All monoclonal antibodies were purchased by BioLegend (San Diego, CA, USA). Reagents used are listed in Online Resource 1. To wash cultured PBMCs, phosphate-buffered saline (PBS) was used. Re-suspension of isolated cells in staining buffer containing PBS + 1% FCS + 0.09% sodium azide followed. Viable cells (0.5–1 × 10^6^ cells) were subsequently incubated with optimally titrated labeled antigen-specific monoclonal antibodies on ice for 30 min. Afterwards, paraformaldehyde (2%) was utilized for cell fixation. Logarithmic amplification was used, while lymphocytes were sub-gated based on forward and side light scatter characteristics. To accurately measure infrequent cell subsets, at least 3 × 10^5^ events within the lymphocyte gate were collected. Both high background and false positive readouts were excluded based on isotype control monoclonal antibodies.

### Flow cytometric viability analysis

To assess viability of PBMCs after thawing, each sample was stained with FICT-conjugated Annexin V (BioLegend, San Diego, CA, USA), which identifies apoptotic cells, per manufacturer’s instructions. Cells were washed twice, and then were re-suspended in Annexin V Binding Buffer. PBMCs were subsequently incubated with optimally titrated Annexin V in the dark for 15 min. Finally, Annexin V Binding Buffer was added in each tube and each sample was analyzed with flow cytometry.

### Flow cytometric intracellular cytokine detection

Intracellular IFN-γ and IL-17 production by PBMCs was measured by flow cytometry utilizing anti-human fluorochrome-conjugated monoclonal antibodies. Briefly, detection of intracellular cytokine production involved use of peridinin chlorophyll protein (PerCP)–conjugated anti-IL-17A (clone BL168) and APC-Cy7-conjugated anti-IFN-γ (clone 4S.B3), obtained by BioLegend (San Diego, CA, USA). Following surface staining and fixation, cells were then permeabilized using Perm/Wash buffers (BD Biosciences). Incubation with optimally titrated antibodies followed. Online Resource 2 summarizes cell subset characterization by surface and intracellular marker staining.

### Statistical analysis

Descriptive statistical measures (mean and standard deviation (SD)) were used to describe cohort’s characteristics. We presented surface-epitope expressing cell fractions as percentages and described them using a mean ± SD. To compare percentages regarding a specific cell subset between GA-treated and -untreated cells, a paired *t*-test or a Wilcoxon signed-rank test was used. Mean values and 95% confidence interval (CI) or median values of difference were used to describe differences between groups, depending on the type of comparison (parametric or non-parametric test used). To correlate changes in cell fractions between various cell sub-populations, Pearson correlation coefficient or Spearman’s rank correlation coefficient was used. To assess the effect of therapy on GA-mediated inhibition of IFN-γ and IL-17 in various cell subsets, a simple linear regression was utilized. A *p* value of ≤ 0.05 was defined as significant. Statistical calculations were executed within Graphpad Prism software.

## Results

The effect of various concentrations of GA on IL-17 and IFN-γ expression in PBMCs isolated by both participants with psoriasis (*n* = 28) and HCs (*n* = 12) was examined. Based on previous in vitro experiments involving GA of the literature, the final concentrations of GA tested were 5, 10, 30, 50, and 100 μg/mL for either 0.5 or 2 h. The strongest effect of GA on cytokine abrogation that was accompanied by cell viability preservation was observed in the presence of 30 μg/mL GA 0.5 h prior stimulation. Viability of cells at this concentration was maintained, while higher concentrations of GA diminished cell viability. The optimal concentration of GA for our experiments was thus considered to be 30 μg/mL (Online Resource 3). This concentration corresponds to that used for in vitro experiments using cell lines in published series [32–34].

**Fig. 1:**
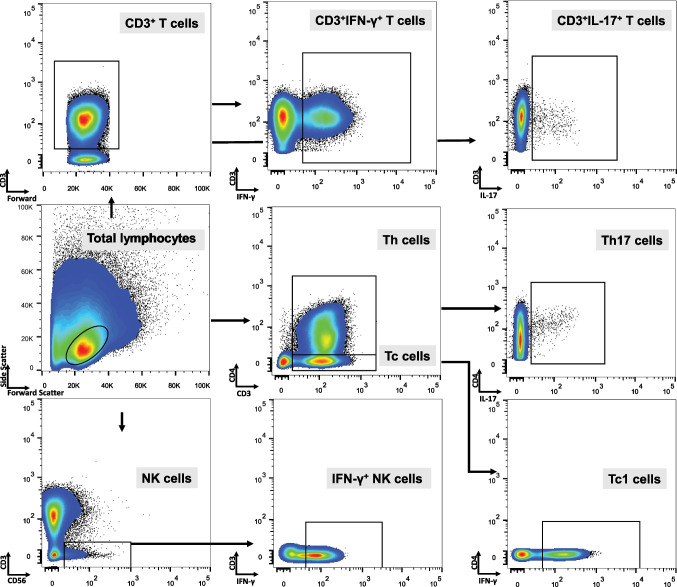
Subdividing strategy as depicted in representative plots from flow cytometry. Total lymphocytes were sub-gated based on forward/side light scatter characteristics. Next, Th (CD3^+^CD4^+^) and Tc (CD3^+^CD4^−^) sub-populations were characterized utilizing CD3-CD4 staining. Furthermore, using CD3-CD56 staining, NK (CD3^−^CD56^+^) cells were also recognized. Within each cell sub-population IL-17 and IFN-γ expression was identified by IL-17 and IFN-γ staining respectively

PBMCs were effectively sub-gated utilizing both surface and intracellular staining. Figure 1 presents gating strategy used to analyze our data. Cell viability for each sample used was assessed after thawing of cells. Percentage of cells that were stained with Annexin V, which identifies apoptotic cells, was consistently ≤ 10%. GA treatment did not affect cell viability. CD3^+^CD4^+^ and CD3^+^CD4^−^ cell sub-population percentages were not affected by GA treatment. The CD3^+^CD4^+^ and CD3^+^CD4^−^ cell population percentages did not significantly differ between the GA-treated and -untreated PBMCs, as depicted in Online Resource 4.

### GA pre-treatment did not alter fractions of IL-17 or IFN-γ-producing PMA-stimulated PBMC subsets in HCs

We initially evaluated the in vitro effect of GA pre-treatment on the percentage of IL-17 and IFN-γ-producing CD3^+^ (T), CD3^+^CD4^+^ (Th), CD3^+^CD4^−^ (Tc), and CD3^−^CD56^+^ (NK) cell sub-populations of PBMCs isolated from HCs. Co-culture of PMA-activated PBMCs isolated from HCs with GA did not affect neither IL-17 nor IFN-γ production within the CD3^+^ compartment (mean of differences: − 0.09, 95% CI − 0.29 to 0.1 for IL-17 and − 0.7, 95% CI − 6.4 to 5 for IFN-γ) or the CD3^+^CD4^+^ cell compartment (mean of differences: − 0.38, 95% CI − 0.85 to 0.089 for IL-17 and − 0.23, 95% CI − 4.45 to 3.99 for IFN-γ). The percentage of cells expressing IFN-γ within both the Tc and the NK cell compartments also did not differ between GA-treated and -untreated culture conditions.

### GA in vitro pre-treatment diminished fractions of IL-17-producing CD3^+^ and CD3^+^CD4^+^ PMA-stimulated cell subsets in psoriasis patients

**Fig. 2:**
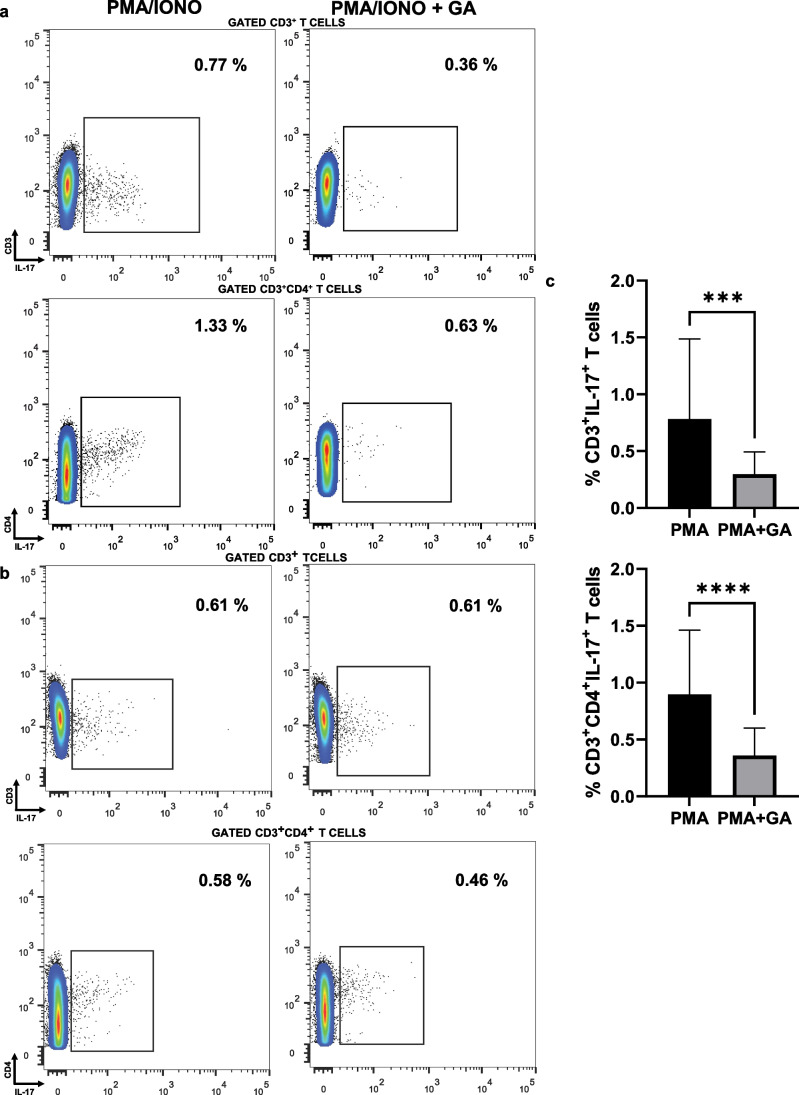
Gallic acid (GA) in vitro pre-treatment diminished fractions of IL-17-producing T (CD3^+^) and Th (CD3^+^CD4^+^) cell subsets that were stimulated by PMA/ionomycin in participants with psoriasis (*n* = 28) and controls (*n* = 12). Isolated peripheral blood mononuclear cells were co-cultured with PMA/ionomycin with or without GA. Cells were analyzed following GA pre-treatment for 0.5 h and PMA and ionomycin stimulation. **a** Representative plots from flow cytometry of GA-treated and -untreated IL-17-producing T and Th cells from patients.** b** Representative plots from flow cytometry of GA-treated and -untreated IL-17-producing T and Th cells from controls. **c** Boxplots (showing mean ± SD) depicting that IL-17-producing T and Th cells were significantly inhibited by in vitro GA pre-treatment in patients. ***p ≤ 0.001, ****p ≤ 0.0001 by Wilcoxon signed-rank test or paired *t*-test

We next explored the effect of GA treatment on the fraction of IL-17-producing PMA-activated cell subsets isolated from participants with psoriasis. Figure 2a,b  presents representative plots from flow cytometry of GA-treated and -untreated cells that were activated with PMA and ionomycin. As shown in Fig. 2c, the percentages of IL-17-producing T and Th cells were significantly inhibited by in vitro GA pre-treatment. Specifically, the sub-population fractions of IL-17^+^ T and Th cells were diminished from mean = 0.78 (SD = 0.70) and mean = 0.89 (SD = 0.56) to mean = 0.29 (SD = 0.19) and mean = 0.35 (SD = 0.24), respectively, which corresponds to a decrease of 63% and 61%, respectively. IL-17 production within the CD3^+^CD4^−^ compartment was not observed.

### GA in vitro pre-treatment diminished fractions of IFN-γ-producing CD3^+^, CD3^+^CD4^+^, CD3^+^CD4^−^, PMA-stimulated cell subsets in psoriasis patients

**Fig. 3:**
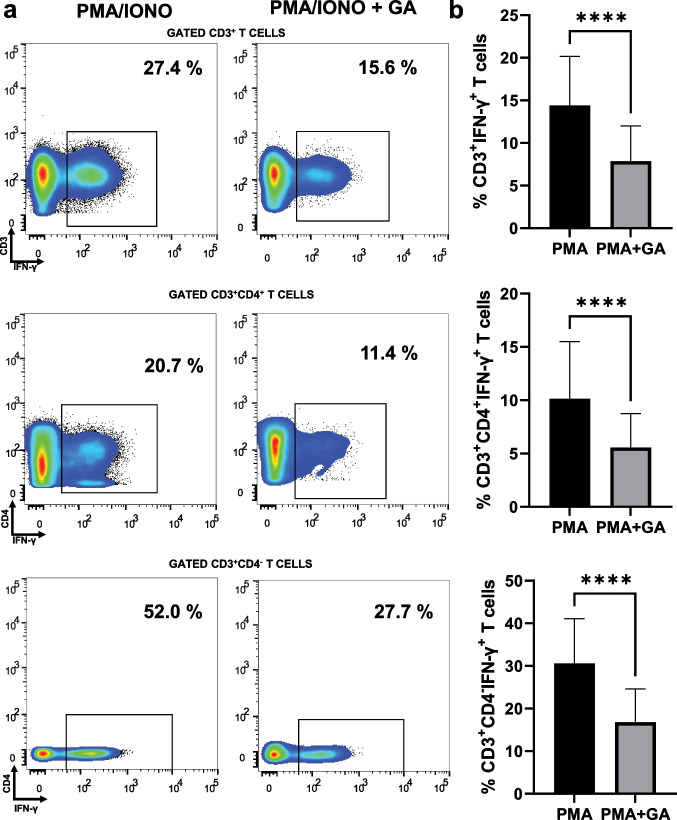
Gallic acid (GA) in vitro pre-treatment diminished fractions of IFN-γ-producing T (CD3^+^), Th (CD3^+^CD4^+^) and Tc (CD3^+^CD4^−^) cell subsets that were stimulated by PMA and ionomycin in participants with psoriasis (*n* = 28). Isolated peripheral blood mononuclear cells were co-cultured with PMA/ionomycin with or without GA. Cells were analyzed following GA pre-treatment for 0.5 h and PMA and ionomycin stimulation. **a** Representative plots from flow cytometry of GA-treated and -untreated IFN-γ-producing T, Th, and Tc cells. **b** Boxplots (showing mean ± SD) depicting that IFN-γ-producing T, Th, and Tc cells were significantly inhibited by in vitro GA pre-treatment. ****p ≤ 0.0001 by Wilcoxon signed-rank test or paired *t*-test

We also investigated the effect of GA treatment on the fraction of IFN-γ-producing PMA-activated cell subsets isolated from participants with psoriasis. Figure 3a presents representative plots from flow cytometry of GA-treated and -untreated cells that were stimulated with PMA and ionomycin. As shown in Fig. 3b , significant inhibition by in vitro GA pre-treatment was observed in the percentages of IFN-γ-producing T (from mean = 14.4 (SD = 5.74) to mean = 7.87 (SD = 4.13)), Th (from mean = 10.14 (SD = 5.37) to mean = 5.55 (SD = 3.17)), and Tc (from mean = 30.62 (SD = 10.44) to mean = 16.78 (SD = 7.84)) cells, compared to the percentages of GA-untreated PMA-activated subsets.

### GA in vitro pre-treatment diminished fraction of IFN-γ-producing CD3^−^CD56^+^, PMA-stimulated cell subsets in individuals with psoriasis

**Fig. 4:**
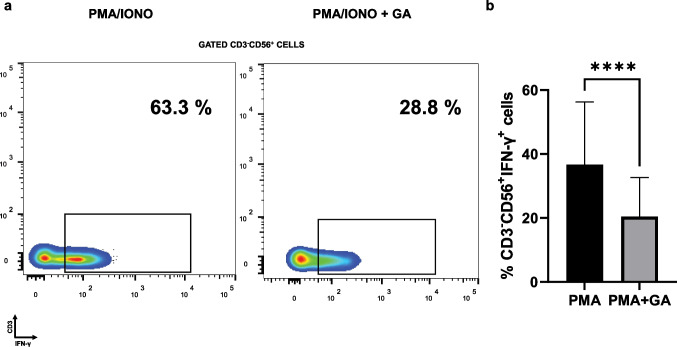
Gallic acid (GA) in vitro pre-treatment diminished the fraction of the IFN-γ-producing ΝΚ (CD3^−^CD56^+^) cell subset that was stimulated by PMA/ionomycin in participants with psoriasis (*n* = 28). Isolated peripheral blood mononuclear cells were co-cultured with PMA/ionomycin with or without GA. Cells were analyzed following GA pre-treatment for 0.5 h and PMA and ionomycin stimulation. **a** Representative plots from flow cytometry of GA-treated and -untreated IFN-γ-producing NK cells. **b** Boxplots (showing mean ± SD) depicting that IFN-γ-producing NK cells were significantly inhibited by in vitro GA pre-treatment, ****p ≤ 0.0001 by Wilcoxon signed-rank test or paired *t*-test

We also investigated the effect of GA-treatment on the frequencies of IFN-γ expressing CD3^*−*^CD56^+^ NK subset in PMA-stimulated PBMCs isolated from participants with. Figure 4a presents representative plots from flow cytometry of a psoriasis sample that was evaluated under GA-treated and -untreated PMA-stimulated conditions for IFN-γ production. As shown in Fig. 4b, IFN-γ production in NK cells that were activated by PMA and ionomycin was significantly inhibited by in vitro GA pre-treatment, from a mean = 36.67 (SD = 19.58) to a mean = 20.42 (SD = 12.21).

### Level of GA-mediated inhibition on Th1 cells was correlated with the inhibition of Th17 and IFN-γ-producing NK cells in PMA-stimulated PBMCs isolated by psoriasis patients

**Fig. 5:**
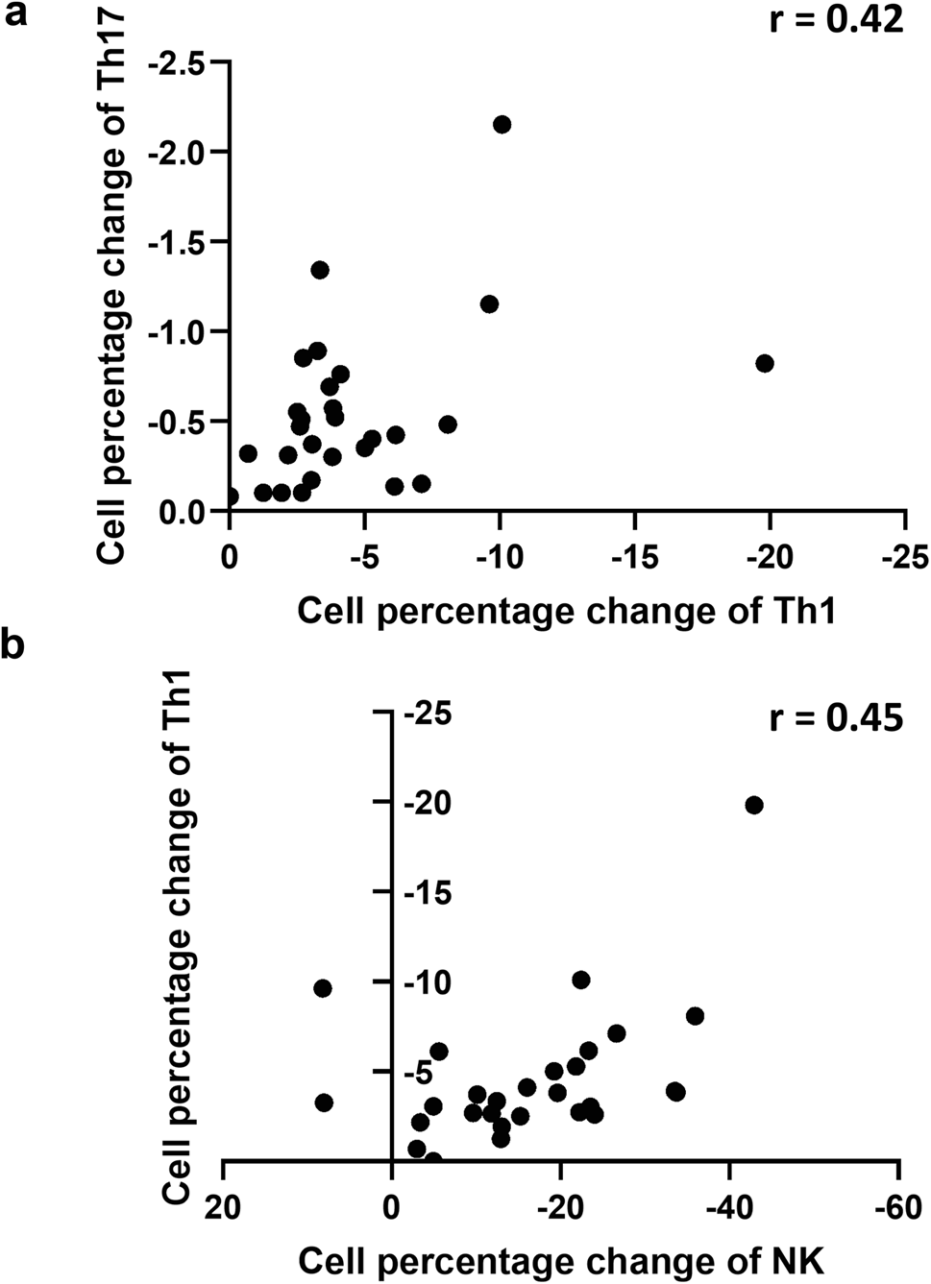
Gallic acid (GA) in vitro pre-treatment diminished percentages of Th17-, Th1-, and IFN-γ-producing NK cell subsets that were stimulated by PMA/ionomycin in participants with psoriasis (*n* = 28). Isolated peripheral blood mononuclear cells were co-cultured with PMA/ionomycin with or without GA. Cells were analyzed following GA pre-treatment for 0.5 h and PMA and ionomycin stimulation. **a** GA-mediated inhibition on the IFN-γ-producing Th cell percentage was correlated with the inhibition of the fraction of IL-17-producing Th cells. **b** GA-mediated inhibition on the IFN-γ-producing Th cell percentage was correlated with the inhibition of the fraction of IFN-γ-producing NK cells

We explored associations between levels of GA pre-treatment-induced inhibition of each cell subset fraction investigated in this study. Between GA-treated and -untreated cell cultures that were activated with PMA and ionomycin, a mean of change values was calculated for Th17, Th1-, Tc1-, and IFN-γ-producing NK cells. As depicted in Fig. 5, change in Th1 cell percentages after GA treatment was significantly correlated with change in percentage of Th17 (Pearson *r* = 0.42, *p* = 0.024) and of percentage of IFN-γ-producing NK cells (Pearson *r* = 0.45, *p* = 0.015). GA’s activity may not accrue by an effect on a specific PBMC sub-population, but rather by a simultaneous inhibitory effect on various pro-inflammatory cell populations.

### GA-mediated inhibition of IFN-γ production in CD3^+^CD4^−^ cells is higher in PBMCs isolated by patients treated with biologic therapies

We investigated whether treatment received during sample collection affected the GA-mediated inhibition of IL-17- or IFΝ-γ-producing PBMC subsets. Participants diagnosed with psoriasis vulgaris were grouped into 3 categories: those naïve to treatment (*n* = 8), those receiving biologic therapy (*n* = 8), and those receiving non-biologic therapy (*n* = 12, DMARDs and apremilast). A simple linear regression was executed to assess the effect of therapy received during sample collection on the change in cell frequency between GA-treated and -untreated conditions for each cell sub-population. The mean cell percentage difference between GA-treated and -untreated cells for each population was used as a dependent variable, while the treatment received was used as independent variable for each simple regression model. Therapy received did not affect the mean difference of Th17-, Th1-, and IFN-γ-producing NK cells between GA-treated and GA-untreated cultures (Online Resource 5). Regarding IFN-γ-producing CD3^+^CD4^−^ cells, cells isolated from patients receiving biologic therapy showed a significantly higher decrease in IFN-γ production following GA in vitro treatment compared to those isolated by patients naïve to therapy for psoriasis vulgaris (Online Resource 5).

## Discussion

To our knowledge, this is the first study to explore the effect of GA supplementation on Th17-, Th1-, and IFN-γ-producing NK cells in patients diagnosed with psoriasis vulgaris. We hereby present a remarkable decrease in the frequencies of the aforementioned PMA-stimulated cell subsets upon in vitro GA exposure. Interestingly, the sharp reduction in IFN-γ-producing cell percentages mediated by GA included Tc cells too. Our in vitro results clearly demonstrate direct effects of GA on pro-inflammatory cytokines, which remarkably are observed in psoriasis patients, but not in HCs. Whether GA affects cytokine production in a disease-specific manner remains to be explored in future studies enrolling both larger cohorts and other disease-control patients. Nevertheless, we are confident that our data are significant, as the inhibitory effect of GA was consistent in all psoriasis patients tested, while lack of such an inhibitory effect was noted in every single healthy control assessed. Our data could be intriguing given that such a ramification noted in psoriasis patients but not in HCs was not evident in our previous in vitro study using the naturally GA-enriched compound of delphinidin.

The GA-mediated decrease on IL-17- and IFN-γ-producing cells provides indications that GA may be a compound of interest in any effort to explore new anti-inflammatory agents against the disease. IL-17 has long been characterized as the crucial cytokine in the pathogenesis of psoriasis [35]. High efficacy anti-IL-17 biologic therapies are used in the management of the disease [36]. The mechanisms responsible for the inhibitory effect of GA on IL-17 production by CD4^+^ cells are not clear. Transcription factor ROR-γt has been identified as a lineage defining factor of Th17 cells [37]. In nasal lavage fluid collected by ovalbumin-induced murine allergic rhinitis murine model, GA treatment suppressed levels of ROR-γt [26]. Furthermore, STAT3 has also been characterized as a Th17 transcription factor [38]. Inhibition of p-STAT3 has been reported to be mediated by GA [25, 29]. Additional to the inhibitory effect of GA on IL-17 production, the compound has been reported to diminish the activity of IL-17-stimulated downstream signal transduction pathways. The cytokine-driven feed-forward loop facilitated in psoriasis is transduced by certain molecules, including NF-κB. Mutations in genes encoding activators of NF-κB have been associated with psoriasis development [39]. Interestingly, GA has been found to suppress NF-κB phosphorylation in LPS-challenged IPEC-J2 cell cultures [27].

We also observed an effect of GA pre-treatment on the percentage of IFN-γ-producing NK cells following PMA stimulation. GA significantly decreased the fraction of CD3^−^CD56^+^IFN-γ^+^ cell subset. NK cells have been suggested to participate in the establishment of the inflammation loop of psoriasis [40]. Specifically, NK cells have been reported to accumulate in psoriatic skin and promote inflammation [41, 42].

Of relevance, recent in vitro evidence suggests that GA inhibits additional immune pathways that are known to be actively involved in the disease. For example, IL-6 has been identified as a key cytokine that participates in pro-inflammatory mechanisms in skin and eventually leads to development of psoriatic skin lesions. In psoriatic skin, dendritic cells produce high levels of IL-6, which promotes escape from Treg suppression [43] and skewing of CD4^+^ T cells towards a Th17 phenotype [44]. GA has been shown to mediate inhibition of IL-6 production in IL-33-activated basophilic KU812 cells in vitro [45]. In another study, poly(GA) reduced IL-6 levels in supernatants of PMA-stimulated THP-1 cells [28]. Furthermore, dendritic cells produce TNF-α [46], a central molecule in the inflammatory milieu of psoriasis, which directly affects keratinocyte gene expression [47]. In murine models, GA treatment has been associated with decreased levels of TNF-α in mouse sera [24].

The effect of GA on immune cell populations has been partly investigated [9]. In a recent in vitro study, GA was shown to enhance Foxp3^+^ cells (iTreg differentiation when C57BL/6 mice spleen-isolated CD4^+^ T cells were co-cultured in the presence of GA with TGF-β and IL-2). Moreover, GA co-culture with iTregs suppressed Teff proliferation compared to cultures where GA was absent. In an in vivo study, intraperitoneal administration of GA in an allograft rejection mouse model expanded Tregs and decreased activated T cells [7]. In a murine model of arthritis, GA decreased local inflammation, and inhibited the in vitro release of neutrophil TNF-a [48]. Furthermore, in monosodium urate crystal stimulated swelling of joints in mice, GA mitigated symptoms and inhibited IL-1β expression [49]. In the human setting, GA treatment of fibroblast-like synoviocytes isolated from patients with rheumatoid arthritis increases the levels of pro-apoptotic caspase-3 and decreases the expression of pro-inflammatory molecules in vitro [50]. Whether GA’s immunoregulating activity could also be exploited for the management of inflammatory joint diseases remains to be seen, but early in vitro or in vivo data suggest that such a probability should not be underestimated.

In our study, GA-mediated inhibition of IFNγ production was significantly higher in CD3^+^CD4^−^ T cells isolated from patients receiving bDMARDs compared to PBMCs isolated from treatment-naïve patients. A synergistic effect of GA with biotechnological drugs has been documented. In a melanoma-bearing mouse model, combination of GA and an IDO inhibitor exerted an in vitro effect in CD8^+^ and Tregs [51]. An in vitro combined effect of GA with doxorubicin was stronger in suppressing growth of DU145 prostate cancer cells [52] and the combined effect was also noted in human squamous carcinoma [53] and glioma cell lines [33]. To our knowledge, our in vitro study is the first to indicate an indirect synergistic effect of GA with a bDMARD. A hypothesis regarding the molecular mechanisms that could explain such a synergism could be formed. While bDMARDs inhibit the end product of the pro-inflammatory feed-forward loop, such as IL-23 or IL-17, GA has been reported to inhibit NF-κB and STAT3, and thus effectively decrease the proportion of pro-inflammatory cells such as the circulating Th17 cells. As shown in this study, GA inhibits the proportion of PBMCs producing IFNγ and IL-17. GA has also been suggested to have high binding affinities with the receptors of INF-a2, IL-4, and IL-6 [54]. Our in vitro approach prevented us from speculative arguments that could exploit this further but future research will shed a light in revealing such a possible synergism.

In our study, GA’s inhibitory effect on PBMC pro-inflammatory subpopulations was significant in patients with psoriasis and not in HCs. Our data cannot provide a mechanistic elucidation for such a finding. We could only hypothesize that the selective effect of GA in activated PBMCs is mediated by STAT3 and/or NF-κB hyperactivation; STAT3 hyperactivation significantly contributes to cytokine production and lesion development in patients with psoriasis [55], and GA is able to inhibit phosphorylation of STAT3. A “STAT3 hyperactivated state” in patients with psoriasis but not in HCs may account for the exerted inhibitory effect of GA and that may also be the case for NF-κB. Both molecules have been targeted in the past for the management of the disease [56]. Such speculative scenarios must be addressed experimentally in vitro and in vivo.

Whether GA could be utilized as a therapeutical agent against psoriasis or as a supplement to conventional treatment needs to be determined in proper in vivo studies and well-designed randomized control clinical trials. Important tackles to overcome relate to the absorption and bioavailability characteristics of GA. A few studies have investigated the absorption and bioavailability characteristics of GA. Orally administered concentrated tea brew (about three times concentrated compared to normal tea brew) in individuals led to a maximum plasma GA concentration of 2 μmol/L after approximately 1 h of tea consumption[57]. The reported relatively low plasma concentration is of limited relevance (up to 80 times lower) to the concentration used in our in vitro study, suggesting that the observed effects on PBMC subsets could result from bioenhanced pharmacologically relevant GA concentrations in order to be of clinical significance. The relatively low plasma concentrations achieved from oral compounds may limit its therapeutic potential. Such limitations have been observed in other diet supplements such as curcumin and led to the development of more bioactive formulas. Repeated dosing [58], usage of phospholipid complexation [59], or colloidal delivery system development [9] as a way to enhance the therapeutic effect of GA have also been investigated with favorable results. Finally, the biological effects of GA may be hampered by the rapid metabolism in humans. Poor stability and solubility may also explain the observed inconsistent oral bioavailability of GA [9].

GA appears to be very safe and significant issues regarding its toxicity have not been raised. Acute toxicity of GA is reported; only enormous doses are administered in mice (≥ 2000 mg/kg) [60]. No adverse events were observed in F344 rats fed with 120 mg/kg/day for 13 weeks [61]. Furthermore, in a murine model of pelvic inflammatory disease, administration of 210 mg/kg had no toxic effect [62]. In another study investigating the toxicity of GA, researchers reported that even high doses of up to 900 mg/kg/day GA for 28 days were not toxic [63]. Based on these results, GA’s administration could be evaluated as clinically relevant, since toxicity is reported only in extremely high doses. Nevertheless, toxicity and safety issues of more bioactive formulas must be assessed.

We consider that our results could constitute the impetus for future in vitro or in vivo studies that assess the pharmaceutical effect of GA in any form—ranging from the dietary intake to orally supplemented GA as a concentrated compound or even to topically applied creams or lotions containing GA alone or in conjunction to other anti-inflammatory agents—on psoriatic lesions.

## Supplementary Information

Below is the link to the electronic supplementary material.

Supplementary file1 (PDF 59 KB)

Supplementary file2 (PDF 57 KB)

Supplementary file3 Online Resource 3 Effect of various concentrations of GA on the viability of isolated PBMCs from patients with psoriasis at 0.5 h and 2 h (n = 5). Presented data represent three independent experiments. Plot shows mean ± SD. (PPTX 56 KB)

Supplementary file4 Online Resource 4 Isolated peripheral blood mononuclear cells were co-cultured with PMA/ionomycin with or without GA. Cells were analyzed following GA pre-treatment for 1 h and PMA/ionomycin stimulation. CD3+CD4+ and CD3+CD4- cells were not affected by GA treatment therapy and frequencies remained relatively unchanged. a Relatively unchanged fractions of CD3+CD4+ and CD3+CD4- T cell populations between GA-treated and -untreated cells in participants with psoriasis (n = 28), as presented in representative plots from flow cytometry. b Boxplot showing no significant difference in mean CD3+CD4+ and CD3+CD4- cell subset percentages between GA-treated and -untreated cells. ns p >0.05, by paired t-test. (PPTX 107 KB)

Supplementary file5 (DOCX 14 KB)

## Data Availability

The data underlying this article will be shared on reasonable request to the corresponding author.

## References

[1] Chiricozzi A, Romanelli P, Volpe E, Borsellino G, Romanelli M. Scanning the immunopathogenesis of psoriasis [Internet]. Int. J. Mol. Sci. MDPI AG; 2018 [cited 2021 Mar 31]. Available from: https://pubmed.ncbi.nlm.nih.gov/29316717/. 10.3390/ijms19010179 PMC5796128 29316717

[2] Goldminz AM, Au SC, Kim N, Gottlieb AB, Lizzul PF (2013). NF-κB: an essential transcription factor in psoriasis. J Dermatol Sci.

[3] Tsiogkas SG, Mavropoulos A, Dardiotis E, Zafiriou E, Bogdanos DP. A sharp decrease of Th17, CXCR3 +-Th17 and Th17.1 in peripheral blood is associated with an early anti-IL-17-mediated clinical remission in psoriasis. Clin Exp Immunol. England; 2022. 10.1093/cei/uxac069 PMC9585551 35925616

[4] Kamali AN, Noorbakhsh SM, Hamedifar H, Jadidi-Niaragh F, Yazdani R, Bautista JM (2019). A role for Th1-like Th17 cells in the pathogenesis of inflammatory and autoimmune disorders. Mol Immunol England.

[5] Johnson-Huang LM, Suárez-Fariñas M, Pierson KC, Fuentes-Duculan J, Cueto I, Lentini T (2012). A single intradermal injection of IFN-γ induces an inflammatory state in both non-lesional psoriatic and healthy skin. J Invest Dermatol.

[6] Tsiogkas SG, Mavropoulos Α, Skyvalidas DN, Patrikiou E, Ntavari N, Daponte AI, et al. Delphinidin diminishes in vitro interferon-γ and interleukin-17 producing cells in patients with psoriatic disease. Immunol Res. 2021 10.1007/s12026-021-09251-y 34825313

[7] Hyun KH, Gil KC, Kim SG, Park S-Y, Hwang KW (2019). Delphinidin chloride and its hydrolytic metabolite gallic acid promote differentiation of regulatory T cells and have an anti-inflammatory effect on the allograft model. J Food Sci United States.

[8] Kern M, Fridrich D, Reichert J, Skrbek S, Nussher A, Hofem S (2007). Limited stability in cell culture medium and hydrogen peroxide formation affect the growth inhibitory properties of delphinidin and its degradation product gallic acid. Mol Nutr Food Res.

[9] Yang K, Zhang L, Liao P, Xiao Z, Zhang F, Sindaye D (2020). Impact of gallic acid on gut health: focus on the gut microbiome, immune response, and mechanisms of action. Front Immunol.

[10] AL Zahrani NA, El-Shishtawy RM, Asiri AM. Recent developments of gallic acid derivatives and their hybrids in medicinal chemistry: a review. Eur J Med Chem [Internet]. Elsevier Masson SAS; 2020;204:112609. Available from: 10.1016/j.ejmech.2020.112609. 10.1016/j.ejmech.2020.112609 32731188

[11] Choubey S, Varughese LR ache., Kumar V, Beniwal V. Medicinal importance of gallic acid and its ester derivatives: a patent review. Pharm Pat Anal. 2015;4:305–15. 10.4155/ppa.15.14 26174568

[12] Kumar N, Goel N. Phenolic acids: Natural versatile molecules with promising therapeutic applications. Biotechnol Reports [Internet]. Elsevier B.V.; 2019;24:e00370. Available from: 10.1016/j.btre.2019.e00370. 10.1016/j.btre.2019.e00370 PMC6734135 31516850

[13] Badhani B, Sharma N, Kakkar R. Gallic acid: a versatile antioxidant with promising therapeutic and industrial applications. RSC Adv [Internet]. Royal Society of Chemistry; 2015;5:27540–57. Available from: 10.1039/C5RA01911G.

[14] Cho EJ, Yokozawa T, Rhyu DY, Kim HY, Shibahara N, Park JC (2003). The inhibitory effects of 12 medicinal plants and their component compounds on lipid peroxidation. Am J Chin Med.

[15] Dhingra MS, Dhingra S, Chadha R, Singh T, Karan M. Design, synthesis, physicochemical, and pharmacological evaluation of gallic acid esters as non-ulcerogenic and gastroprotective anti-inflammatory agents. Med Chem Res [Internet]. 2014;23:4771–88. Available from: 10.1007/s00044-014-1041-x.

[16] Lone SH, Rehman SU, Bhat KA. Synthesis of gallic-acid-1-phenyl-1H-[1,2,3]triazol-4-yl methyl esters as effective antioxidants. Drug Res (Stuttg). Germany; 2017;67:111–8. 10.1055/s-0042-118860 27824429

[17] Nenadis N, Lazaridou O, Tsimidou MZ (2007). Use of reference compounds in antioxidant activity assessment. J Agric Food Chem United States.

[18] Schlesier K, Harwat M, Böhm V, Bitsch R (2002). Assessment of antioxidant activity by using different in vitro methods. Free Radic Res England.

[19] Locatelli C, Filippin-Monteiro FB, Creczynski-Pasa TB. Alkyl esters of gallic acid as anticancer agents: A review. Eur J Med Chem [Internet]. Elsevier; 2013;60:233–9. Available from: 10.1016/j.ejmech.2012.10.056. 10.1016/j.ejmech.2012.10.056 23291333

[20] Inoue M, Suzuki R, Sakaguchi N, Li Z, Takeda T, Ogihara Y (1995). Selective induction of cell death in cancer cells by gallic acid. Biol Pharm Bull Japan.

[21] You BR, Park WH. Gallic acid-induced lung cancer cell death is related to glutathione depletion as well as reactive oxygen species increase. Toxicol Vitr an Int J Publ Assoc with BIBRA. England; 2010;24:1356–62. 10.1016/j.tiv.2010.04.009 20417267

[22] Kaur M, Velmurugan B, Rajamanickam S, Agarwal R, Agarwal C (2009). Gallic acid, an active constituent of grape seed extract, exhibits anti-proliferative, pro-apoptotic and anti-tumorigenic effects against prostate carcinoma xenograft growth in nude mice. Pharm Res.

[23] Inoue M, Sakaguchi N, Isuzugawa K, Tani H, Ogihara Y (2000). Role of reactive oxygen species in gallic acid-induced apoptosis. Biol Pharm Bull Japan.

[24] Hsiang CY, Hseu YC, Chang YC, Kumar KJS, Ho TY, Yang HL. Toona sinensis and its major bioactive compound gallic acid inhibit LPS-induced inflammation in nuclear factor-κB transgenic mice as evaluated by in vivo bioluminescence imaging. Food Chem [Internet]. Elsevier Ltd; 2013;136:426–34. Available from: 10.1016/j.foodchem.2012.08.009. 10.1016/j.foodchem.2012.08.009 23122080

[25] Yang R, Li Z, Zou Y, Yang J, Li L, Xu X (2021). Gallic acid alleviates neuropathic pain behaviors in rats by inhibiting P2X7 receptor-mediated NF-κB/STAT3 signaling pathway. Front Pharmacol.

[26] Fan Y, Piao CH, Hyeon E, Jung SY, Eom JE, Shin HS, et al. Gallic acid alleviates nasal inflammation via activation of Th1 and inhibition of Th2 and Th17 in a mouse model of allergic rhinitis. Int Immunopharmacol [Internet]. Elsevier; 2019;70:512–9. Available from: 10.1016/j.intimp.2019.02.025. 10.1016/j.intimp.2019.02.025 30884431

[27] Cai L, Wei Z, Zhao X, Li Y, Li X, Jiang X. Gallic acid mitigates LPS-induced inflammatory response via suppressing NF-κB signalling pathway in IPEC-J2 cells. J Anim Physiol Anim Nutr (Berl). 2021;1–9. 10.1111/jpn.13612 34288130

[28] Zamudio-Cuevas Y, Andonegui-Elguera MA, Aparicio-Juárez A, Aguillón-Solís E, Martínez-Flores K, Ruvalcaba-Paredes E (2021). The enzymatic poly(gallic acid) reduces pro-inflammatory cytokines in vitro, a potential application in inflammatory diseases. Inflammation Inflammation.

[29] Zhang T, Ma L, Wu P, Li W, Li T, Gu R (2019). Gallic acid has anticancer activity and enhances the anticancer effects of cisplatin in non-small cell lung cancer A549 cells via the JAK/STAT3 signaling pathway. Oncol Rep.

[30] Zhou G, Kong WS, Li ZC, Xie RF, Yu TY, Zhou X. Effects of Qing Chang suppository powder and its ingredients on IL-17 signal pathway in HT-29 cells and DSS-induced mice. Phytomedicine [Internet]. Elsevier GmbH; 2021;87:153573. Available from: 10.1016/j.phymed.2021.153573. 10.1016/j.phymed.2021.153573 34052543

[31] Skyvalidas D, Mavropoulos A, Tsiogkas S, Dardiotis E, Liaskos C, Mamuris Z, et al. Curcumin mediates attenuation of pro-inflammatory interferon γ and interleukin 17 cytokine responses in psoriatic disease, strengthening its role as a dietary immunosuppressant. Nutr Res [Internet]. Elsevier Inc.; 2020;75:95–108. Available from: 10.1016/j.nutres.2020.01.005. 10.1016/j.nutres.2020.01.005 32114280

[32] Zhao B, Hu M (2013). Gallic acid reduces cell viability, proliferation, invasion and angiogenesis in human cervical cancer cells. Oncol Lett Greece.

[33] Yang J-T, Lee I-N, Chen C-H, Lu F-J, Chung C-Y, Lee M-H, et al. Gallic acid enhances the anti-cancer effect of temozolomide in human glioma cell line via inhibition of Akt and p38-MAPK pathway. Processes. 2022.

[34] Sourani ZM, Pourgheysari BP, Beshkar PM, Shirzad HP, Shirzad MM (2016). Gallic acid inhibits proliferation and induces apoptosis in lymphoblastic leukemia cell line (C121). Iran J Med Sci Iran.

[35] Liang Y, Sarkar MK, Tsoi LC, Gudjonsson JE, Arbor A, Arbor A, et al. Psoriasis: a mixed autoimmune and autoinflammatory disease. 2018;1–8. 10.1016/j.coi.2017.07.007 PMC5705427 28738209

[36] Kanda N (2021). Psoriasis: pathogenesis, comorbidities, and therapy updated. Int J Mol Sci.

[37] Capone A, Volpe E. Transcriptional regulators of T helper 17 cell differentiation in health and autoimmune diseases. Front Immunol. 2020;11. 10.3389/fimmu.2020.00348 PMC7080699 32226427

[38] Foley JF. STAT3 regulates the generation of Th17 Cells. Sci STKE [Internet]. American Association for the Advancement of Science; 2007;2007:tw113–tw113. Available from: 10.1126/stke.3802007tw113.

[39] Jordan CT, Cao L, Roberson EDO, Duan S, Helms CA, Nair RP (2012). Rare and common variants in CARD14, encoding an epidermal regulator of NF-kappaB, in psoriasis. Am J Hum Genet.

[40] Gardiner CM, Dunphy S. NK cells and psoriasis. J Biomed Biotechnol. 2011;2011. 10.1155/2011/248317 PMC3114545 21687543

[41] Ottaviani C, Nasorri F, Bedini C, de Pità O, Girolomoni G, Cavani A (2006). CD56brightCD16(-) NK cells accumulate in psoriatic skin in response to CXCL10 and CCL5 and exacerbate skin inflammation. Eur J Immunol Germany.

[42] Kastelan M, PrpićMassari L, Gruber F, Zamolo G, Zauhar G, Coklo M (2004). Perforin expression is upregulated in the epidermis of psoriatic lesions. Br J Dermatol England.

[43] Goodman WA, Levine AD, Massari JV, Sugiyama H, McCormick TS, Cooper KD (2009). IL-6 signaling in psoriasis prevents immune suppression by regulatory T cells. J Immunol.

[44] Acosta-Rodriguez EV, Napolitani G, Lanzavecchia A, Sallusto F (2007). Interleukins 1beta and 6 but not transforming growth factor-beta are essential for the differentiation of interleukin 17-producing human T helper cells. Nat Immunol United States.

[45] Liu KYP, Hu S, Chan BCL, Wat ECL, Lau CBS, Hon KL (2013). Anti-inflammatory and anti-allergic activities of pentaherb formula, moutan cortex (Danpi) and gallic acid. Molecules.

[46] Lowes MA, Chamian F, Abello MV, Fuentes-Duculan J, Lin S-L, Nussbaum R (2005). Increase in TNF-alpha and inducible nitric oxide synthase-expressing dendritic cells in psoriasis and reduction with efalizumab (anti-CD11a). Proc Natl Acad Sci U S A.

[47] Chiricozzi A, Guttman-Yassky E, Suárez-Fariñas M, Nograles KE, Tian S, Cardinale I (2011). Integrative responses to IL-17 and TNF-α in human keratinocytes account for key inflammatory pathogenic circuits in psoriasis. J Invest Dermatol United States.

[48] Jiang D xun, Zhang M hua, Zhang Q, Chen Y shan, Ma W jing, Wu W peng, et al. Influence of gallic acid on porcine neutrophils phosphodiesterase 4, IL-6, TNF-α and rat arthritis model. J Integr Agric [Internet]. Chinese Academy of Agricultural Sciences; 2015;14:758–64. Available from: 10.1016/S2095-3119(14)60824-8.

[49] Lin Y, Luo T, Weng A, Huang X, Yao Y, Fu Z (2020). Gallic acid alleviates gouty arthritis by inhibiting NLRP3 inflammasome activation and pyroptosis through enhancing Nrf2 signaling. Front Immunol.

[50] Yoon CH, Chung SJ, Lee SW, Park YB, Lee SK, Park MC. Gallic acid, a natural polyphenolic acid, induces apoptosis and inhibits proinflammatory gene expressions in rheumatoid arthritis fibroblast-like synoviocytes. Jt Bone Spine [Internet]. Elsevier Masson SAS; 2013;80:274–9. Available from: 10.1016/j.jbspin.2012.08.010. 10.1016/j.jbspin.2012.08.010 23058179

[51] Liu H, Gao H, Chen C, Jia W, Xu D, Jiang G (2022). IDO Inhibitor and gallic acid cross-linked small molecule drug synergistic treatment of melanoma. Front Oncol.

[52] Chen HM, Wu YC, Chia YC, Chang FR, Hsu HK, Hsieh YC, et al. Gallic acid, a major component of Toona sinensis leaf extracts, contains a ROS-mediated anti-cancer activity in human prostate cancer cells. Cancer Lett [Internet]. Elsevier Ireland Ltd; 2009;286:161–71. Available from: 10.1016/j.canlet.2009.05.040. 10.1016/j.canlet.2009.05.040 19589639

[53] Rajagopalan R, Jain SK, Trivedi P (2019). Synergistic anti-cancer activity of combined 5-fuorouracil and gallic acid-stearylamine conjugate in a431 human squamous carcinoma cell line. Trop J Pharm Res.

[54] Yadav DK, Khan F, Negi AS (2012). Pharmacophore modeling, molecular docking, QSAR, and in silico ADMET studies of gallic acid derivatives for immunomodulatory activity. J Mol Model.

[55] Calautti E, Avalle L, Poli V. Psoriasis: a STAT3-centric view. Int J Mol Sci. Switzerland; 2018;19. 10.3390/ijms19010171 PMC5796120 29316631

[56] Andrés RM, Montesinos MC, Navalón P, Payá M, Terencio CM (2013). NF-κB and STAT3 inhibition as a therapeutic strategy in psoriasis: in vitro and in vivo effects of BTH. J Invest Dermatol.

[57] Shahrzad S, Aoyagi K, Winter A, Koyama A, Bitsch I (2001). Pharmacokinetics of gallic acid and its relative bioavailability from tea in healthy humans. J Nutr United States.

[58] Ferruzzi MG, Lobo JK, Janle EM, Cooper B, Simon JE, Wu Q-L (2009). Bioavailability of gallic acid and catechins from grape seed polyphenol extract is improved by repeated dosing in rats: implications for treatment in Alzheimer’s disease. J Alzheimers Dis.

[59] Bhattacharyya S, Ahammed SM, Saha BP, Mukherjee PK (2013). The gallic acid-phospholipid complex improved the antioxidant potential of gallic acid by enhancing its bioavailability. AAPS PharmSciTech.

[60] Variya BC, Bakrania AK, Madan P, Patel SS. Acute and 28-days repeated dose sub-acute toxicity study of gallic acid in albino mice. Regul Toxicol Pharmacol [Internet]. Elsevier; 2019;101:71–8. Available from: 10.1016/j.yrtph.2018.11.010. 10.1016/j.yrtph.2018.11.010 30465803

[61] Niho N, Shibutani M, Tamura T, Toyoda K, Uneyama C, Takahashi N, et al. Subchronic toxicity study of gallic acid by oral administration in F344 rats. Food Chem Toxicol an Int J Publ Br Ind Biol Res Assoc. England; 2001;39:1063–70. 10.1016/s0278-6915(01)00054-0 11527565

[62] Li Y, Yang Q, Shi Z, Zhou M, Yan L, Li H, et al. The anti-inflammatory effect of Feiyangchangweiyan capsule and its main components on pelvic inflammatory disease in rats via the regulation of the NF-*κ*B and BAX/BCL-2 pathway. Evidence-Based Complement Altern Med [Internet]. Hindawi; 2019;2019:9585727. Available from: 10.1155/2019/9585727. 10.1155/2019/9585727 PMC6595388 31312226

[63] Variya BC, Bakrania AK, Madan P, Patel SS. Acute and 28-days repeated dose sub-acute toxicity study of gallic acid in albino mice. Regul Toxicol Pharmacol [Internet]. 2019;101:71–8. Available from: https://www.sciencedirect.com/science/article/pii/S0273230018303027.. 10.1016/j.yrtph.2018.11.010 30465803

