# Supplementary material for: Gallic acid diminishes pro-inflammatory interferon-γ- and interleukin-17-producing sub-populations in vitro in patients with psoriasis
Source: Immunol Res. 2023 Feb 9;71(3):475–87. doi: 10.1007/s12026-023-09361-9 (PMC10185625; doi:10.1007/s12026-023-09361-9)
Supplement: Supplementary file 1 — Supplementary file1 (PDF 59 KB) [file 12026_2023_9361_MOESM1_ESM.pdf]

Online Resource1 List of monoclonal antibodies used in flow cytometry

Antibody	Fluorochrome	Source	Clone
Mouse anti-human CD3	FICT	BioLegend	UCHT1
Mouse anti-human CD56	PE	BioLegend	HCD56
IL-17A	PerCP	BioLegend	BL168
Mouse anti-human CD4	Pe-Cy7	BioLegend	RPA-T4
IFN-γ	APC-Cyanine7	BioLegend	4S.B3

IL-17A: interleukin-17A, IFN-γ: interferon-γ
