# Supplementary material for: Gallic acid diminishes pro-inflammatory interferon-γ- and interleukin-17-producing sub-populations in vitro in patients with psoriasis
Source: Immunol Res. 2023 Feb 9;71(3):475–87. doi: 10.1007/s12026-023-09361-9 (PMC10185625; doi:10.1007/s12026-023-09361-9)
Supplement: Supplementary file 2 — Supplementary file2 (PDF 57 KB) [file 12026_2023_9361_MOESM2_ESM.pdf]

Online Resource 2 Characterization of cell sub-populations by staining markers

Phenotype	Cell population
$CD3^+$	T cells
$CD3^+CD4^+$	Th cells
$CD3^+CD4^-$	Tc cells
$CD3^+CD4^+IL\text{-}17A^+$	Th17 cells
$CD3^+CD4^+IFN\text{-}\gamma^+$	Th1 cells
$CD3^+CD4^-IL\text{-}17A^+$	Tc17 cells
$CD3^+CD4^-IFN\text{-}\gamma^+$	Tc1 cells
$CD3^-CD56^+$	NK cells
$CD3^+CD56^+$	NKT cells

Th: T helper; Tc: T cytotoxic; NK: Natural killer; NKT: Natural killer T
