# Supplementary figures and images for: Gallic acid diminishes pro-inflammatory interferon-γ- and interleukin-17-producing sub-populations in vitro in patients with psoriasis
Source: Immunol Res. 2023 Feb 9;71(3):475–87. doi: 10.1007/s12026-023-09361-9 (PMC10185625; doi:10.1007/s12026-023-09361-9)

## Slide 1
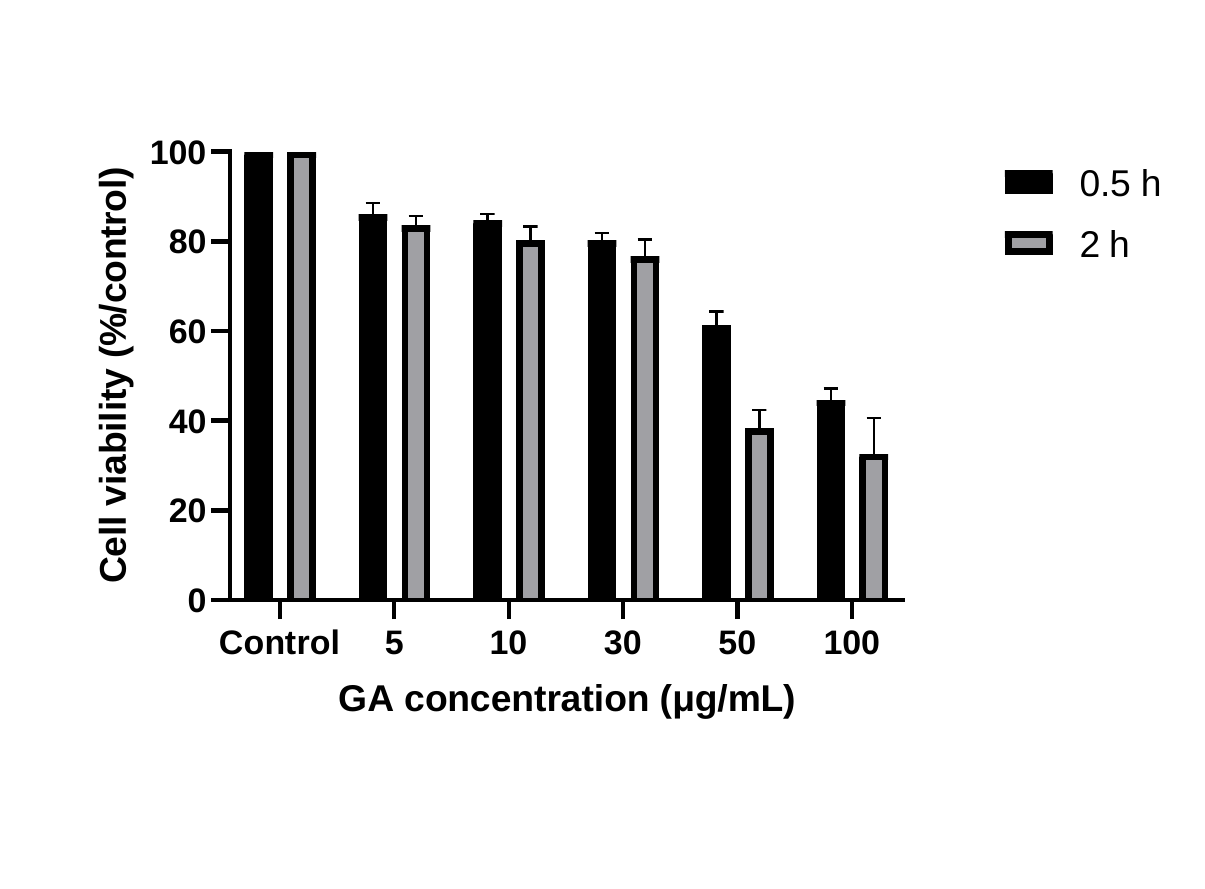

Supplement: Supplementary file 3 — Supplementary file3 Online Resource 3 Effect of various concentrations of GA on the viability of isolated PBMCs from patients with psoriasis at 0.5 h and 2 h (n = 5). Presented data represent three independent experiments. Plot shows mean ± SD. (PPTX 56 KB) [file 12026_2023_9361_MOESM3_ESM.pptx]

## Slide 1
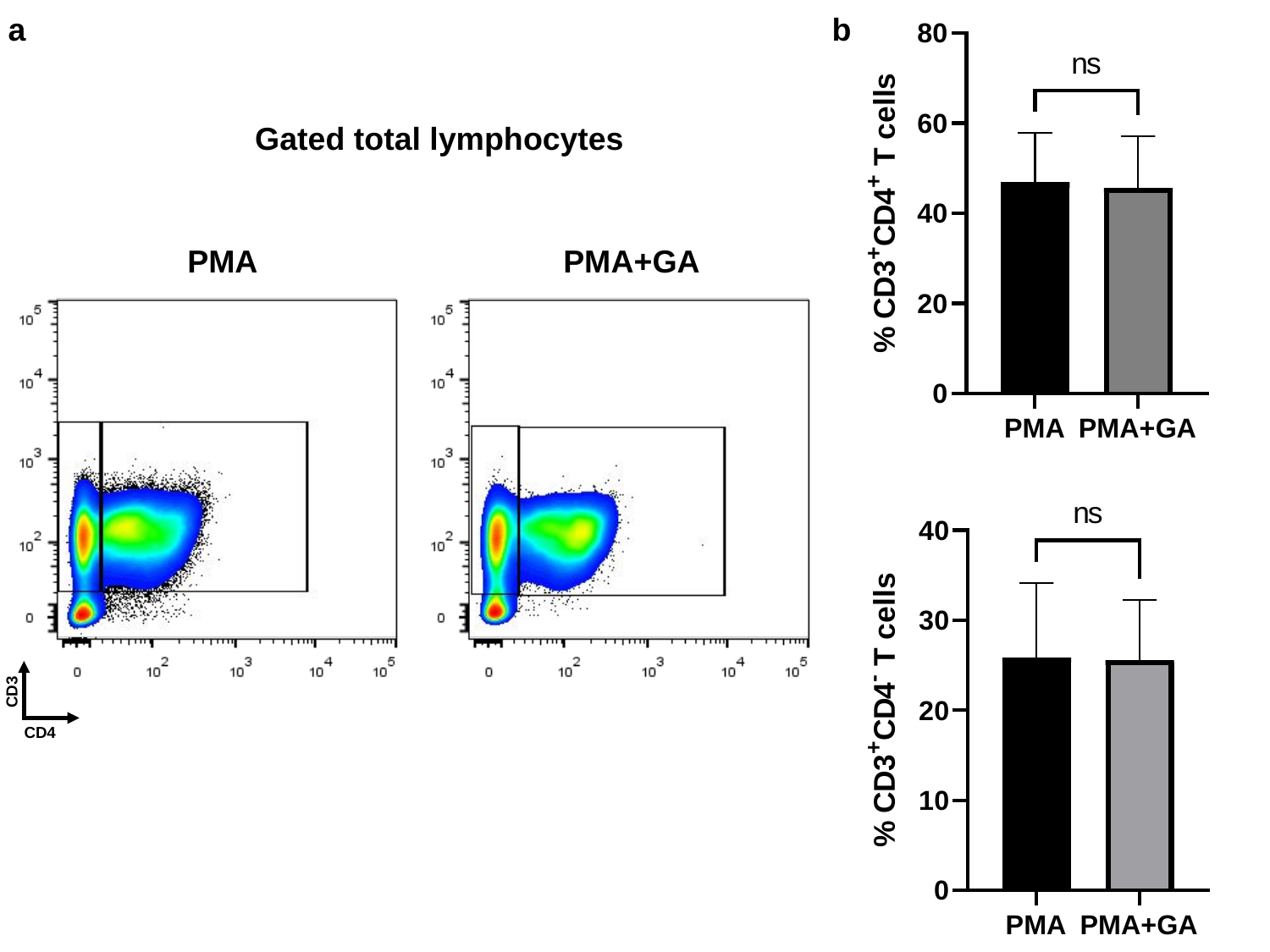

a
b
Gated total lymphocytes
PMA
PMA+GA
CD3
CD4

Supplement: Supplementary file 4 — Supplementary file4 Online Resource 4 Isolated peripheral blood mononuclear cells were co-cultured with PMA/ionomycin with or without GA. Cells were analyzed following GA pre-treatment for 1 h and PMA/ionomycin stimulation. CD3+CD4+ and CD3+CD4- cells were not affected by GA treatment therapy and frequencies remained relatively unchanged. a Relatively unchanged fractions of CD3+CD4+ and CD3+CD4- T cell populations between GA-treated and -untreated cells in participants with psoriasis (n = 28), as presented in representative plots from flow cytometry. b Boxplot showing no significant difference in mean CD3+CD4+ and CD3+CD4- cell subset percentages between GA-treated and -untreated cells. ns p >0.05, by paired t-test. (PPTX 107 KB) [file 12026_2023_9361_MOESM4_ESM.pptx]
