# Supplementary material for: Gallic acid diminishes pro-inflammatory interferon-γ- and interleukin-17-producing sub-populations in vitro in patients with psoriasis
Source: Immunol Res. 2023 Feb 9;71(3):475–87. doi: 10.1007/s12026-023-09361-9 (PMC10185625; doi:10.1007/s12026-023-09361-9)
Supplement: Supplementary file 5 — Supplementary file5 (DOCX 14 KB) [file 12026_2023_9361_MOESM5_ESM.docx]

Online Resource 4 Simple linear regressions assessing the effect of treatment on mean GA-mediated differences of cell subsets.

| Simple Linear Regression Number | Dependent Variable | Independent Variable (Therapy group/Reference group) | β coefficient | 95% CI | p value |
| --- | --- | --- | --- | --- | --- |
| 1 | Th17_Dif | biologics/naïve | 0.051 | -4.08 to 0.51 | 0.22 |
|  |  | NB/naïve | 0.292 | -0.12 to 0.71 | 1.4 |
| 2 | Th1_Dif | biologics/naïve | -3.284 | -7.01 to 0.44 | 1.8 |
|  |  | NB/naïve | 0.468 | -2.93 to 3.87 | 0.28 |
| 3 | Tc1_Dif | biologics/naïve | -8.725 | -15.38 to -2.07 | **0.01** |
|  |  | NB/naïve | -0.844 | -6.91 to 5.22 | 0.77 |
| 4 | IFN-γ producing NK_DIf | biologics/naïve | -4.173 | -16.59 to 8.24 | 0.49 |
|  |  | NB/naïve | 5.817 | -5.52 to 17.15 | 0.3 |

Th: T helper; Tc: T cytotoxic; NK: Natural killer
